# Abdominal wall complications following renal transplantation in adult recipients – factors associated with interventional management in one unit

**DOI:** 10.1186/s12893-019-0468-x

**Published:** 2019-01-21

**Authors:** Ngee-Soon Lau, Nima Ahmadi, Deborah Verran

**Affiliations:** Department of Transplant Surgery, Royal Prince Alfred Hospital, Sydney, New South Wales Australia

**Keywords:** Wound complications, Renal transplantation, Negative pressure wound therapy

## Abstract

**Background:**

Abdominal wall surgical site complications following renal transplantation can be challenging to manage. A sub-group of these recipients will require operative management or advanced wound care such as negative pressure wound therapy (NPWT). The aim of this study was to determine if there were any preoperative, intraoperative and postoperative characteristics in our recipients’ cohort which were associated with the requirement for such interventions.

**Methods:**

A retrospective review of medical records was performed for all recipients who sustained abdominal wall complications following renal transplantation at our centre from 2006 to 2016.

**Results:**

A total of 64/828 recipients (7.7%) had abdominal wall complications. The mean weight for these patients was 84.9 kg (±16.6 kg) and the mean body mass index was 30.2 (±5.1). Forty-five recipients (70%) had a superficial wound dehiscence while nine (14%) had a complete fascial dehiscence. Operative intervention was required in 13/64 patients (20%) and was more likely to be required in the presence of a fascial dehiscence (9/9, 100%) or a wound collection (10/31, 32%) (*p* < 0.001, *p* = 0.021). NPWT was used in 17/64 patients (27%) and was more commonly required in patients with diabetes mellitus (10/24, 42%), a complete fascial dehiscence (5/9, 56%) or evidence of infection (16/44, 36%) (*p* = 0.039, *p* = 0.034, *p* = 0.008).

**Conclusions:**

The requirement for either operative management or the use of NPWT in the management of abdominal wall complications following renal transplantation in our experience was more common in recipients with diabetes mellitus, and in the setting of either complete fascial dehiscence, abdominal wall wound collections and/ or infection.

## Background

Abdominal wall complications are common following renal transplantation with published rates varying from 10.5–15.4% [1–5]. The range of reported complications encompasses the spectrum of superficial wound dehiscence, complete fascial dehiscence, wound infections, and perigraft fluid collections [6]. The underlying cause of these various surgical site complications is felt to be multi-factorial in nature. The known risk factors include: recipient factors such as age, obesity, diabetes mellitus, smoking and malnutrition; surgical factors such as surgical technique, method of closure, surgical complications; and renal transplant-specific risk factors such as immunosuppressive therapy, delayed graft function and the requirement for dialysis post-transplant [6].

The management of abdominal wall complications following renal transplantation can be challenging and time consuming and contributes to prolonging the recovery time of the recipient. There are limited data on the length of time to complete wound healing in this population but one study found that as many as 21% of patients still had incomplete healing at 5 weeks post-transplant [3]. Although the majority of recipients can be managed with less invasive modalities such as simple dressings and antibiotics when appropriate, the literature reports that many as 25% of recipients will also require further intervention including operative management [1, 3, 4]. Hence, if it were possible to identify in advance the recipients who are at high-risk for sustaining a significant abdominal wall complication, then this could assist with both their perioperative and postoperative management.

In addition, a sub-group of recipients may benefit from advanced wound management techniques such as negative pressure wound therapy (NPWT). NPWT is a well-accepted technique for the management of complex soft tissue wounds and is now used for problematic wounds in a range of surgical settings [7]. However, it is only relatively recently that NPWT has been used in recipients with wound complications following renal transplantation and the reported experience remains limited [8]. The main utility of NPWT appears to be via shortening the time to healing either as a bridge to delayed primary closure of the superficial abdominal wall or as a way to hasten healing by secondary intention [9, 10].

In this study, we describe our experience of managing abdominal wall complications following renal transplantation in adult recipients in a single centre and examine the underlying characteristics of recipients who required either operative intervention and/or NPWT. In this way, we aim to understand the patterns in our use of either operative intervention and/or NPWT as well as to identify which subgroup of recipients are more likely to require these treatment modalities. This may allow us to better understand our clinical decision-making and thereby work in the future to improve our clinical outcomes.

## Methods

### Recipients

All adult recipients with abdominal wall complications were initially identified from a departmental database of all postoperative complications following renal transplantation from 2006 to 2016. The inclusion criteria for this study were recipients with any type of wound and/or abdominal wall surgical site complication which occurred in the first 30 days following renal transplantation, who required further management. Recipients who underwent combined liver-kidney transplantation, recipients with isolated lymphoceles and/or perigraft collections (including those discovered on radiological imaging) and recipients who had delayed abdominal wall complications such as incisional hernias were all excluded. We also excluded patients who were lost to follow up or who were transferred to a different transplant service. A total of 16 patients that were otherwise eligible were excluded in this way (8 incisional hernias, 2 combined transplants, 6 subfascial collections).

A retrospective review of the electronic and hard-copy medical records was then performed. The following data were obtained: recipient demographics (age, sex, weight and body mass index), medical comorbidities (presence of diabetes mellitus, peripheral vascular disease, smoking status, aetiology of chronic kidney disease, type of dialysis and years of dialysis), operative details (type of donor, side of transplant and type of skin closure) and postoperative details (presence of delayed graft function, need for postoperative dialysis, time to abdominal wall healing, abdominal wall complications, requirement for further procedures). Particular attention was paid to whether recipients required either an operative intervention and/or NPWT and for what type of abdominal wall complication whether it be a wound collection, evidence of surgical site infection, or a superficial or complete fascial wound dehiscence. Wound collections were diagnosed using clinical findings and supporting radiology if required; infection was a microbiological diagnosis based on positive wound culture results; and wound dehiscence was clinically defined as superficial (skin and subcutaneous tissue only) or complete (fascial dehiscence).

The primary end points were the requirement for either operative intervention or NPWT. Secondary end points were the presence of superficial or deep fascial dehiscence, time to complete healing, and the duration of NPWT.

This study was approved by the Sydney Local Health District Ethics Review Committee (Royal Prince Alfred Hospital, Sydney, Australia).

### Methods

Renal transplantation is well established at our institution with an average of 100 kidney transplants each year performed by five transplant surgeons. This includes transplantation from deceased donors following either circulatory or brain death and living donors. A Rutherford Morison incision and standard extraperitoneal approach is used to expose the right or left iliac vessels for the vascular anastomoses and the ureter is reimplanted using a stented Lich-Gregoir ureteroneocystostomy. Prophylactic antibiotics are administered at the time of induction of anaesthesia and a Jackson-Pratt drain is placed routinely. The incision is closed in layers in a standard manner either using skin clips, an absorbable subcuticular suture or multiple simple-interrupted sutures. The indwelling catheter is removed on postoperative day 5 and the surgical drain removed when the output over 24 h is less than 30 ml. Immunosuppression usually consists of induction with basiliximab or antithymocyte globulin followed by maintenance with a glucocorticoid, mycophenolate mofetil and a calcineurin inhibitor. Notably, during the study period, our local protocol was not to use mammalian target of rapamycin (mTOR) inhibitors during the first 30 days after transplant. On discharge from hospital, recipients are routinely followed up in an outpatient clinic setting for review of the surgical site and abdominal wall and for ongoing medical and immunosuppressant management. Abdominal wall complications are identified either during routine inpatient review or follow up reviews in the outpatient clinic. Wound collections are diagnosed using ultrasound or computed tomography and wound infections are diagnosed by both clinical signs of infection (redness, tenderness, purulent discharge) and bacteriological culture.

The abdominal wall complications are managed according to the severity of the problem as well as the resulting impact on the recipient. Management options include and may utilise: simple dressings, oral or intravenous antibiotics, radiological drainage, operative intervention or NPWT. Operative intervention is required in the setting of complete fascial dehiscence, uncontrolled abdominal wall sepsis or abdominal wall collections not amenable to percutaneous drainage. Depending on the circumstances, NPWT may be initiated either in hospital or in the outpatient clinic. NPWT is utilised in wounds that wound benefit from healing by secondary intention either due to infection, or wound dehiscence. Management decisions were under the direction of the treating transplant surgeon and in medically complex recipients also involved renal physician input. Prolonged NPWT was managed by serial dressing changes carried out twice weekly in the outpatient clinic along with regular wound review with subsequent modification of the dressing regimen as required. NPWT was ceased when complete granulation had occurred and the wound small enough to be treated with simple dressings. Following an abdominal wall complication, all recipients were followed up regularly as an outpatient until wound healing was confirmed.

### Statistical analysis

Data was analysed using SPSS statistical analysis software (version 24; SPSS Inc., Chicago, Illinois). Normality was assessed by examining histograms and the Shapiro-Wilk Test. Patients who required operative intervention or NPWT were compared to patients that did not, and patients who had a superficial dehiscence were compared to those with a complete dehiscence. Variables were analysed as appropriate using an independent-samples t-test, Mann-Whitney U test or Pearson’s chi-square test. Results were considered significant if *p* < 0.05.

## Results

Between 2006 and 2016, 828 renal transplants were performed of which 64 recipients were identified as having sustained an abdominal wall complication (7.7%). For the recipients with an abdominal wall wound complication, the mean age was 55 (range 25–72) and there were more males (40/64, 63%) than females. The mean weight was 84.9 kg (95% CI: 80.7–89.0 kg) and the mean body mass index (BMI) fell into the overweight category at 30.2 (95% CI, 28.8–31.5).

Overall, the commonest type of wound complication was a superficial wound dehiscence which occurred in 45/64 (70%) recipients while 9/64 (14%) recipients had a complete wound dehiscence involving the fascia (Table 1). The remaining 10/64 recipients had no evidence of dehiscence but had a superficial type of wound complication;3/10 had a wound infection without evidence of superficial collection, 6/10 had a superficial wound collection (haematoma or seroma) without infection and one had both a wound infection and a collection.

**Table 1:** Sub-group analysis for patients with superficial or complete wound dehiscence

		Type of wound dehiscence		
		Superficial dehiscence (*n* = 45)	Complete dehiscence (*n* = 9)	*p*-value
Age		53.80 ± 11.14	59.11 ± 4.31	*p* = 0.269
BMI		30.08 ± 4.89	32.04 ± 6.48	*p* = 0.470
Years of dialysis		5.04 ± 3.73	4.50 ± 3.64	*p* = 0.798
Glucose (mmol/L)		6.40 ± 1.91	6.58 ± 3.15	*p* = 0.588
Duration of NPWT (days)		40.50 ± 25.80	58.40 ± 36.90	*p* = 0.244
Time to healing (days)		45.69 ± 32.12	37.40 ± 25.81	*p* = 0.882
BMI	Quartile 1	9 (22.0%)	0 (0%)	*p* = 0.266
	Quartile 2	9 (22.0%)	3 (42.9%)	
	Quartile 3	10 (24.4%)	3 (42.9%)	
	Quartile 4	13 (31.7%)	1 (14.3%)	
Sex	Male	30 (66.7%)	4 (44.4%)	*p* = 0.208
	Female	15 (33.3%)	5 (55.6%)	
Type of dialysis	Predialysis	2 (4.5%)	1 (11.1%)	*p* = 0.238
	Haemodialysis	39 (88.6%)	6 (66.7%)	
	Peritoneal dialysis	3 (6.8%)	2 (22.2%)	
Smoker		10/29 (34.5%)	3/6 (50%)	*p* = 0.474
Diabetes mellitus		18/45 (40.0%)	4/8 (50%)	*p* = 0.597
Peripheral vascular disease		5/45 (11.1%)	4/9 (44.4%)	***p*** **= 0.014**
Type of donor	Living	7 (15.6%)	2 (22.2%)	*p* = 0.855
	Brain death	29 (64.4%)	5 (55.6%)	
	Cardiac death	9 (20.0%)	2 (22.2%)	
Immuno-suppression	Tacrolimus^a^	35/45 (77.8%)	6/8 (75%)	*p* = 0.863
	Cyclosporin^b^	10/55 (22.2%)	2/8 (25%)	
Delayed graft function		21/45 (46.7%)	5/9 (55.6%)	*p* = 0.626
Post-operative dialysis		19/45 (42.2%)	5/9 (55.6%)	*p* = 0.462
Infection		32/45 (71.1%)	8/9 (88.9%)	*p* = 0.267
Wound collection		17/45 (37.8%)	7/9 (77.8%)	***p*** **= 0.027**
Operative intervention		3/45 (6.7%)	9/9 (100%)	***p*** **< 0.001**
NPWT		11/45 (24.4%)	5/9 (55.6%)	*p* = 0.062

^a^Tacrolimus based regimen: basiliximab induction followed by maintenance with tacrolimus, a glucocorticoid and mycophenolate mofetil

^b^Cyclosporin based regimen: as above but using cyclosporin instead of tacrolimus

Bold typeface was used to distinguish significant (*p*<0.05) results

A subgroup analysis of the recipients according to the degree of wound dehiscence, revealed that the recipients who sustained a complete deep fascial dehiscence tended to be older (59 vs. 54 years) and had a higher BMI (32 vs. 30), although these did not reach statistical significance (*p* = 0.269, *p* = 0.470) (Table 1). In addition, further analysis of the preoperative, operative and postoperative factors, revealed that the presence of documented peripheral vascular disease (PVD) and the presence of a superficial wound collection were related to the rates of complete fascial dehiscence (*p* = 0.014, *p* = 0.027) (Table 1). The other risk factors of weight, smoking, diabetes, delayed graft function, immunosuppressant regimen and infection were not found to be associated with the type of dehiscence in the abdominal wall (Table 1). Also, the rates of operative intervention were far higher for the patients with a complete fascial dehiscence versus a superficial dehiscence (9/9, 100% vs 3/45, 7%; *p* < 0.001). Further, the use of NPWT was twice as common in the complete fascial dehiscence group, but this did not reach statistical significance. (5/9, 56% vs 11/45, 24%; *p* = 0.062). Finally, the presence of a superficial abdominal wall collection was also significantly related to the need for operative intervention with 10/13 (77%) patients who required an operation also found to have a collection (*p* = 0.021). Otherwise, recipient factors, operative factors and postoperative factors were not significantly related to the need for operative intervention.

An operative intervention was required in 13/64 recipients (20%). The types of operative procedures undertaken were either washout and debridement of the superficial abdominal wall (7/13), or a primary repair of the deep fascia with or without a reinforcing mesh (6/13) (Table 2). Mesh was only utilised in the setting of fascial dehiscence without significant infection or contamination, where resuturing of the fascia alone to gain closure was not technically possible. At the end of these operative procedures the superficial wound was either closed primarily (7/13, 64%) or NPWT was deployed (6/13, 46%). In addition, NPWT was commenced in another 11 recipients who had problems with the superficial abdominal wall outside of the operating room (Table 2). Hence, a total of 17/64 (27%) patients were managed with NPWT. However, the majority of recipients (40/64, 63%) with abdominal wall complications did not require either an operative intervention or initiation of NPWT. Drainage of a superficial wound collection was undertaken in 15/40 of these recipients, either using radiological guidance (9/15, 60%) or at the bedside (6/15, 40%). Finally, 25/40 patients did not require intervention of any kind and were treated with antibiotics in the presence of infection or simple dressings if appropriate.

**Table 2:** Interventions required in patients with wound complications following kidney transplantation

		Number of patients (*n* = 64)
Operative intervention	Washout and debridement	7
	Repair of fascia – primary repair	4
	Repair of fascia – primary repair reinforced with synthetic mesh	2
	**Total**	**13 (20.3%)**
NPWT	Applied on ward^a^	**11 (17.2%)**
Other	Drainage of superficial collection – radiological guidance	9
	Drainage of superficial collection – at bedside	6
	**Total**	**15 (23.4%)**
No intervention		**25 (39.1%)**

^a^6 additional NPWT systems were placed at the time of operative intervention to give a total of 17 patients (26.6%)

Bold typeface was used to distinguish significant (*p*<0.05) results

A further subgroup analysis was undertaken to identify if there were any differences for the recipients with abdominal wall complications who also required NPWT versus those who did not (Table 3). The mean BMI in patients who required NPWT tended to be higher (32 vs 29), and there was a trend towards significance (*p* = 0.058). Furthermore, there was a trend for the use of NPWT to be required in patients with a BMI in the upper two quartiles (*p* = 0.051). However, it was a history of diabetes that emerged as the most significant risk factor for the requirement for NPWT (10/24, 42%, *p* = 0.039) and recipients who required NPWT also had a significantly higher preoperative blood sugar level (8.2 vs 6.0, *p* = 0.021). Finally, evidence of infection at the time of a wound complication also emerged as a significant risk factor with 16/44 (36%) requiring the use of NPWT (*p* = 0.008).

**Table 3:** Sub-group analysis for patients requiring NPWT

		NPWT		
		Yes (*n* = 17)	No (*n* = 47)	p-value
Age		54.71 ± 9.45	54.85 ± 0.73	*p* = 0.642
BMI		32.45 ± 4.93	29.40 ± 4.94	*p* = 0.058
Years of dialysis		4.53 ± 3.00	5.26 ± 4.06	*p* = 0.746
Glucose (mmol/L)		8.17 ± 2.99	5.96 ± 1.69	***p*** **= 0.021**
Duration of NPWT (days)		44.75 ± 29.68	N/A	N/A
Time to healing (days)		70.36 ± 34.33	32.93 ± 25.02	***p*** **= 0.001**
BMI	Quartile 1	0 (0.0%)	12 (29.3%)	*p* = 0.051
	Quartile 2	6 (42.9%)	7 (17.1%)	
	Quartile 3	3 (21.4%)	12 (29.3%)	
	Quartile 4	5 (35.77%)	10 (24.4%)	
Sex	Male	11 (64.7%)	29 (61.7%)	*p* = 0.826
	Female	6 (35.3%)	18 (38.3%)	
Type of dialysis	Predialysis	1 (5.9%)	3 (6.5%)	*p* = 0.744
	Haemodialysis	14 (82.4%)	34 (73.9%)	
	Peritoneal dialysis	2 (11.8%)	9 (19.6%)	
Smoker		7/14 (50.0%)	9/27 (33.3%)	*p* = 0.300
Diabetes mellitus		10/17 (58.8%)	14/46 (30.4%)	***p*** **= 0.039**
Peripheral vascular disease		4/17 (23.5%)	6/47 (12.8%)	*p* = 0.295
Type of donor	Living	1 (5.9%)	9 (19.1%)	*p* = 0.106
	Brain death	14 (82.4%)	25 (53.2%)	
	Cardiac death	2 (11.8%)	13 (27.7%)	
Delayed graft function		9/17 (52.9%)	21/47 (44.7%)	*p* = 0.559
Post-operative dialysis		8/17 (47.1%)	20/47 (42.6%)	*p* = 0.748
Infection		16/17 (94.1%)	28/47 (59.6%)	***p*** **= 0.008**
Wound collection		8/17 (47.1%)	23/47 (48.9%)	*p* = 0.894

Bold typeface was used to distinguish significant (*p*<0.05) results

After a wound complication, the median time to complete healing was 33.5 days (range: 1–153 days) and the median time to complete healing in patients requiring operative intervention was slightly longer than in patients where a non-operative approach was taken (40.5 days vs 31.5 days). In patients requiring NPWT, the median length of NPWT was 35 days (range 16–119 days) and the median time to overall wound healing was 73 days (range: 19–153 days). For patients in which NPWT was not required, the median time to healing was 29 days (range: 1–116 days).

## Discussion

In this study, our rate of abdominal wall complications at 7.7% was lower than published rates, which vary from 10.5–21% [1–5]. Several of these reported studies, however included subfascial perigraft collections and lymphoceles in the cohort with wound complications, which this study instead excluded from further analysis. For example, Fockens et al. [5] found a wound complication rate of 12/108 (11%) in their cohort however 8/12 (66%) of these cases included recipients with lymphoceles, whilst Hernandez et al. [1] reported a wound complication rate of 92/870 (10.5%), but again a large proportion of these recipients 51/92 (55%) were those who also had lymphoceles. Three further studies revealed wound complication rates of 15.4, 21 and 11.7% respectively which included wound infections and wound dehiscence but excluded subfascial collections [2–4].

One of the factors which is felt to contribute to the high rates of wound complications in renal transplant recipients is the requirement for the use of immunosuppressant medications such as mycophenolate mofetil, cyclosporine, calcineurin inhibitors (CNI) and mammalian target of rapamycin (mTOR) inhibitors [11]. In particular, caution has been advised for the use of mTOR inhibitors in obese recipients as this has been associated with increased rates of all wound complications when compared to CNI-based regimens [12, 13]. At our centre during the study time period, mTOR inhibitors were not used for immunosuppression in the first month post operatively which may also be a factor in the slightly lower rates of abdominal wall complications seen in this particular cohort.

In our recipients with wound complications, the average weight was 84.9 kg and the average BMI was 30.2. In 2014, 27.9% of Australians were obese (BMI > 30) and 35.5% overweight (BMI 25–30) [14]. In our study, the finding that BMI tended to be higher in patients with complete wound dehiscence who then required operative management or NPWT, is consistent with the published literature where BMI is a well-established risk factor for wound complications after kidney transplantation. A higher BMI has been found to be associated with not only fascial dehiscence and superficial breakdown [15, 16] but also surgical site infections [17–19], and hence the rates of wound complications overall [20–22]. The underlying cause for this is most likely multi-factorial but is thought to be related to decreased collagen deposition, increased tension on the fascial edges, high microbial loads on moist skin and in skin folds, impaired vascular supply and altered glucose metabolism [23]. Also, Taha et al. [24] in a prospective study found that mid-abdominal circumference was a significant predictor for wound complications after kidney transplant suggesting that it may be central adiposity and abdominal fat rather than BMI itself that causes the increased risk in wound complications. This may have been a factor in our cohort.

The high rates of diabetes mellitus (DM) in our study population at 38%, is higher than the Australian national prevalence of 5.1% [14]. However, DM was also the commonest underlying cause for patients’ chronic kidney disease (17/64, 27%). In Australia, end stage renal failure is caused by DM in 35% of patients [25]. Despite this, we found that DM was not associated with abdominal wall complications such as a complete fascial dehiscence or the need for an operative intervention. DM is well known to impair wound healing, especially in the setting of non-healing foot ulcers [26]. However, in the setting of abdominal wounds, DM has not been isolated as a risk factor for dehiscence [27, 28]. There is also disagreement in the literature about the role of DM in being a risk factor for post renal transplant surgical complications with one study of 270 renal transplant recipients demonstrating no significant difference in postoperative complications between patients with and without DM [29]. Another study of 419 renal transplants showed that DM was a risk factor for overall surgical complications as well as specifically for wound collections on multivariate analysis, however not for overall wound complications, wound infections or wound dehiscence [4]. In the present study, we found that DM was related to the requirement for the use of NPWT. The precise reason for this is unclear, however we postulate that these patients were identified by clinicians as patients whose wounds were unlikely to heal and thus were selected for the use of NPWT. NPWT is well established as superior to conventional treatment for wounds after diabetic foot amputation and this may explain the use of NPWT in our diabetic recipients [30, 31].

Operative intervention was not commonly required in our recipients with a rate of 13/64 (20%) patients, and this was similar to published rates. Hernandez et al. [1] found that 23/92 (25%) patients with wound complications required surgical repair. Other similar retrospective studies had rates of 6/54 (11%) [2], 2/12 (17%) [5], 5/26 (19%) [32], and 50/199 (25%) [3]. In our recipients, operative intervention was significantly related to the presence of a wound collection or a complete fascial dehiscence. This is thought to reflect the magnitude and severity of these less common abdominal wall complications. Other risk factors, in our study, however, were not found to be significantly related. By contrast, in a study of 199 patients, Roine et al. [3] found that reintervention was significantly related to the preoperative risk factors of patient age and BMI. However, lymphoceles were included as wound complications in that particular study, which may partly explain the discrepancy between this and our results. Similarly, in a study of 419 renal transplants, Barba et al. [4] found that grade III complications requiring surgical intervention were independently related to recipient BMI. In our recipients, operative intervention was required for a variety of reasons including fascial dehiscence as well as infected abdominal wall collections. The combination of this and the relatively low numbers of recipients may explain why we were unable to demonstrate similar findings in our recipients.

NPWT is well established in the management of complex wounds, particularly in the context of diabetic foot infections, sternal wounds, and complex soft tissue wounds of the extremities [7]. NPWT has also been used to manage infected laparotomy wounds with some success by reducing length of hospital stay and promoting faster wound healing despite a lack of large-scale high quality trials [33]. However, the use of NPWT in the management of wounds after renal transplant is not well described. Our cohort of 17 patients is the largest detailed series reported to date. In the literature, two case series describing 11 cases in total have described the use of NPWT for infected, non-healing wounds after renal transplant [9, 10]. The first describes the use of NPWT for wound infections with superficial wound dehiscence in nine consecutive patients after renal transplant. The median duration of NPWT in this study was 9 days (range 3–30) [9]. This was shorter than the median of 35 days in the present study. The second case series describes two cases where NPWT was similarly used to manage wounds with superficial dehiscence after renal transplant followed by secondary wound closure at day 14 and day 21 respectively [10]. Three others case reports have described the use of NPWT for the management of either urine leaks or lymphoceles [34–36], and one as a prophylactic measure onto a clean surgical incision [37]. The infrequent use of NPWT, which in our centre was required in 2% of the recipients, may be a factor in the relative under reporting of the use of this modality in renal transplant recipients and hence why there is a lack of data on efficacy. However, in our population, NPWT was more commonly used in recipients either with diabetes mellitus or those with evidence of infection. This trend is likely explained by the expectation of treating surgeons that contaminated wounds or wounds in patients with risk factors for poor healing are better treated using this technique.

In this study, we found that our recipients required NPWT for a longer duration than has been previously described (median 35 days, range 16–119 days). Similar published case series to date however, only describe the use of NPWT for superficial wound dehiscence and infection [9, 10, 36], whereas we commonly use NPWT for not only superficial dehiscence and infection but also as an adjunct to operative intervention after complete fascial dehiscence. In addition, our recipients who required NPWT had a median time to healing of 73 days, which was longer than patients not requiring NPWT at 29 days. By comparison, in the setting of infected laparotomy wounds, NPWT has been found to decrease the time to healing. Zhen et al. [38], in a study of 130 patients found that NPWT significantly decreased the mean time to healing (8.1 vs 18.5 days). As such, we would expect NPWT to shorten the time to healing in our patients. Our practice, however, is to use NPWT for the highest risk wounds that are already expected to have a prolonged healing time, and this may be acting as a selection bias. Additionally, our practice is to continue NPWT until complete wound granulation rather than to utilise secondary suture closure after adequate granulation which may also explain our longer NPWT durations and times to healing.

In respect to patient comorbidities relating to abdominal wall complications and fascial dehiscence, peripheral vascular disease (PVD) was the only patient factor found to have a significant association. This is supported by a study of 441 renal transplant recipients which found that there was a trend towards increasing odds ratio for those with PVD and developing a surgical site infection [39]. Rather than PVD independently causing fascial dehiscence, this may be acting as a marker for the overall condition of these patients which is a factor in the healing of the abdominal wall.

The main limitation of this study is that it is a retrospective cohort analysis of a sub-group of patients with wound complications. Due to limitations with the data available in the unit database, a more comprehensive analysis of the far larger cohort of patients without wound complications was not possible. However, this allowed for a more thorough analysis to be performed of this particular sub-group. As such, we have identified some of the risk factors associated with these complications as well as gained an understanding of the time it takes to gain abdominal wall healing once NPWT is deployed. Finally, regarding the use of operative intervention and NPWT, management decisions were left to the discretion of individual surgeons which may result in a selection bias. However, we believe this still provides valuable information about the group of patients in which operative intervention or NPWT was felt to be required.

## Conclusions

Wound complications are common following kidney transplantation and can be challenging to manage. Operative intervention is occasionally required, particularly in recipients with associated superficial wound collections or complete fascial wound dehiscence. In addition, NPWT appears to be a useful modality for managing the open wounds in the surgical site of the abdominal wall of recipients with risk factors for poor healing. In the largest reported series to date, we have demonstrated the utility of NPWT in renal transplant recipients with a high BMI, diabetes, or concurrent wound infections and as an adjunct to the management of complete fascial dehiscence.

## Abbreviations

BMI: body mass index
CNI: calcineurin inhibitor
DM: diabetes mellitus
mTOR: mammalian target of rapamycin
NPWT: negative pressure wound therapy
PVD: peripheral vascular disease
